# Effect of different wavelengths and powers of laser on surface topography and biofilm removal from titanium implants: an in vitro study

**DOI:** 10.1186/s12903-025-07212-7

**Published:** 2025-12-04

**Authors:** Paul Ashraf Sedrak, Ahmed Adel Abdel Hakim, Josep Arnabat Dominguez, Nermeen Abd Elsalam Rady

**Affiliations:** 1 Faculty of Dentistry, Alexandria University, Champolion St, Azarita, Alexandria 21527 Egypt; 2 Department of Prosthodontics, Faculty of Dentistry, Alexandria University, Champolion St., Azarita, Alexandria 21527 Egypt; 3 Department of Oral Surgery, Faculty of Medicine and Health Sciences, University of Barcelona, Campus de Bellvitge, L’Hospitalet de Ll, Barcelona, 08907 Spain

**Keywords:** Dental implants, Peri-implantitis, Dental laser, Er, Cr:YSGG, Er:YAG, Nd:YAG, Diode laser, Surface topography, Biofilm removal, Scanning electron microscope

## Abstract

**Background:**

This in vitro study aimed to evaluate the effect of using different wavelengths and powers of laser on the surface topography of titanium implants, and to investigate their efficacy in removal of the biofilm complex from the implant surface.

**Methods:**

Ten titanium implants, consisting of five new and five failed implants, were randomized and divided into five separate test groups; (Group 1) Erbium Chromium: Yttrium Scandium Gallium Garnet (Er, Cr: YSGG) 2780 nm, (Group 2) Erbium-doped: Yttrium Aluminum Garnet (Er: YAG) 2940 nm, (Group 3) Neodymium-doped: Yttrium Aluminum Garnet (Nd: YAG) 1064 nm, (Group 4) Diode 940 nm, and (Group 5) Diode 445 nm. Each test group consisted of two implants; one new and one failed implant. A total of 160 implant sites were irradiated. Each area was scanned using Scanning Electron Microscope (SEM) prior to and following laser irradiations. A descriptive analysis was conducted by summarizing the data in terms of frequencies and percentages. Pearson Chi Square test and Fisher’s Exact test were used for comparison between different laser type and laser power intensities. The significance level was set at *P* < .05.

**Results:**

Within the parameters under investigation, both Er, Cr: YSGG and Er: YAG lasers displayed no to minimal alterations in surface topography across the different power intensities. Nd: YAG and Diode lasers showed more evident alterations at high power intensities; with Nd: YAG resulting the most prominent damage to the implant surface. Regarding efficacy in removal of biofilm, Er, Cr: YSGG and Er: YAG lasers consistently exhibited positive results across all different power intensities under investigation. In comparison, Nd: YAG and Diode lasers showed inferior efficacy in biofilm removal at low power intensities with significant power-dependent improvements.

**Conclusions:**

Er, Cr: YSGG and Er: YAG lasers present superior implant decontamination potential without causing notable implant surface alterations. Diode (940 nm) laser can be used at low power intensities without causing detrimental effects. Nd: YAG and Diode (445 nm) lasers are able to disrupt the biofilm complex but can induce more evident implant surface damage.

**Trial registration:**

This is not a human subject research.

## Background

Success of dental implants fundamentally hinges on the complex relationship between the implant surface and the surrounding tissues, a phenomenon epitomized by the concept of osseointegration [1]. The term osseointegration was first described by Brånemark et al. as bone-to‐implant contact on the light microscopic level [2]. Later, Albrektsson and Sennerby defined osseointegration as, “a direct functional and structural connection between living bone and the surface of a load‐carrying implant” [3]. Despite the many advancements in implant material and designs aimed at optimizing osseointegration and achieving biocompatibility, the longevity of implants remains hindered by various complications; one of the most significant being peri-implantitis [4]. According to the World Workshop on the Classification of Periodontal and Peri-Implant Diseases and Conditions (2018), peri‐implantitis is defined as a plaque‐associated pathological condition occurring in tissues around dental implants, that arises subsequent to inflammation of the peri‐implant mucosa and is mainly characterized by progressive loss of implant-supporting bone [5]. Peri-implantitis treatments include various non-surgical and surgical approaches [6]. One of the most widely employed treatment for peri-implantitis is mechanical debridement of the implant surface and the peri-implant area to reduce inflammation by physical removal of the microbial plaque [7, 8]. Other forms of non-surgical treatment included the use of Chlorhexidine gluconate in the form of local irrigation for bacterial count reduction [9, 10]. Antibiotic therapy has also been used in adjunct to surgical and non-surgical procedures to benefit from its bacteriostatic and bactericidal effects [11, 12]. However, there seems to be lack of evidence regarding a gold standard protocol for treatment of peri-implantitis.

One of the advancements that have emerged in recent years is the use of laser for decontamination of the implant surface and promotion of healing and osseointegration. Evidence shows that Er: YAG laser can be used to remove the inflammatory tissues and decontaminate the implant surface [13, 14]. Er, Cr: YSGG laser exhibits comparable efficacy and mechanism of action to Er: YAG laser [15, 16]. Results from previous studies on both types of Erbium lasers showed significant improvements in clinical parameters including gingival index (GI), plaque index (PI), pocket depth (PD), bleeding on probing (BOP), and clinical attachment level (CAL) [13–16]. Diode laser has also been researched for its applications in management of peri-implant diseases; however contradictory results regarding its efficacy were found [17, 18]. One clinical trial concluded that the adjunctive application of Diode laser did not yield any additional positive clinical and radiographical findings, in comparison with mechanical debridement alone [18]. Results from another study showed more significant improvements in BOP, GI and PD, in comparison with using mechanical debridement alone [19]. Studies evaluating the use of Nd: YAG laser seemed to be limited due to the fact that Nd: YAG laser may provoke damage on the implant surface due to its high penetration potential [20, 21].

Therefore, this study aimed to evaluate the effect of using different wavelengths and power settings of dental laser on surface topography of titanium implants, and to investigate the efficacy of each specific wavelength and power of laser irradiation on removal of the biofilm complex from the surface of the implant. The null hypothesis was that no difference would be found regarding degree of surface alteration or efficacy in biofilm removal using different wavelengths and different power settings of lasers.

## Methods

### Samples preparation

Ten titanium implants (BEGO GmbH & Co. KG, Bremen, Germany) with sandblasted, large grit, acid-etched (SLA) surface treatment constituted the sample of the study. These implants consisted of five new implants with no previous extra or intra-oral use or alteration, and five implants that have previously failed to achieve osseointegration in vivo as a result of peri-implantitis. All failed implants were removed and stored for a period of two weeks prior to laser irradiation.

**Fig. 1:**
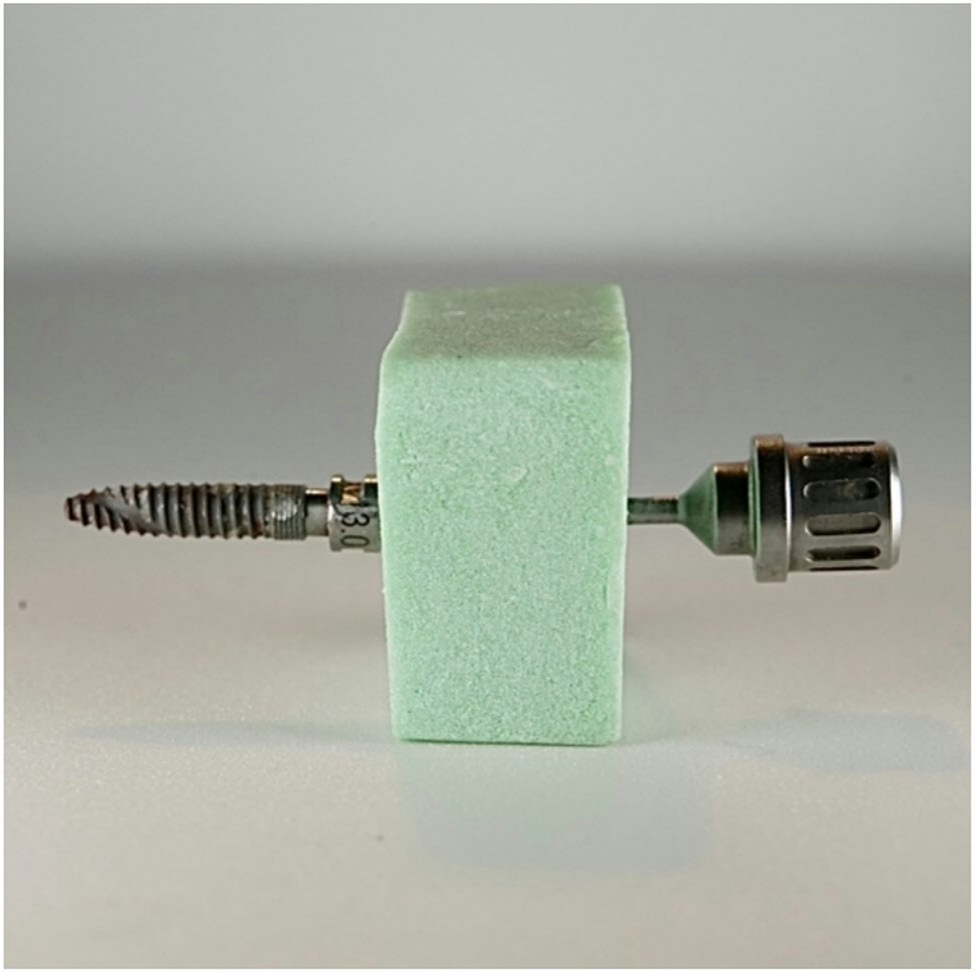
Acrylic block and implant screw used to fixate and remove the implant

A separate custom-made acrylic block was prepared for each implant. The implant’s abutment was fixed in the acrylic block with the thread exposed to be able to accept laser irradiation. To allow ease of fixation and removal of the implants from the acrylic blocks during different steps of the study, a hole was drilled on the side of the block opposite to the abutment of the implant to allow the insertion of an implant screw to fixate and remove the implant body from the abutment situated inside the block and to help obtain the same orientation of the implant between the different steps (Fig. 1).

### Grouping method

**Fig. 2:**
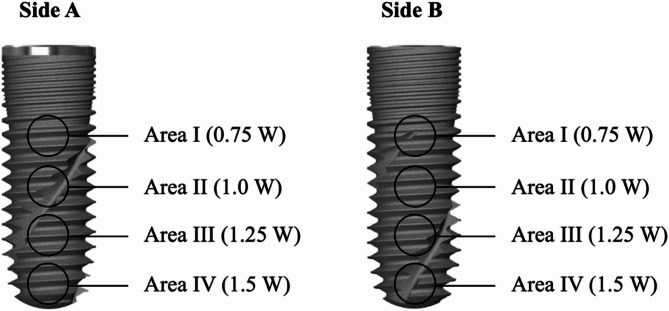
Different irradiation areas using different power settings on two sides of the same implant

The implants were randomized using simple random sampling and divided into five separate test groups using a computer-generated list; (Group 1) Er, Cr: YSGG 2780 nm (Waterlase iPlus, Biolase, San Clemente, U.S.A), (Group 2) Er: YAG 2940 nm (LightWalker, Fotona, Ljubljana, Slovenia), (Group 3) Nd: YAG 1064 nm (LightWalker, Fotona, Ljubljana, Slovenia), (Group 4) Diode 940 nm (EpicX, Biolase, California, USA), and (Group 5) Diode 445 nm (SiroLaser Blue, Dentsply Sirona, Germany). Each test group consisted of two implants; one new and one failed implant. Each implant was then divided into four distinct sides (A, B, C, and D), with each side further alienated horizontally into four areas (I, II, III, and IV). Using a small round bur on a low-speed handpiece, grooves were made on the implant to be able to accurately relocate the different sides and areas of the implant between the steps of the study. Each area (I, II, III, and IV) on every side (A, B, C, and D) was to be irradiated with the power setting under investigation (0.75 W, 1.0 W, 1.25 W, and 1.5 W), respectively (Fig. 2). This allowed 16 irradiation areas per implant and a total of 160 irradiation areas. All designated areas were initially scanned with the aid of SEM (JSM-IT200, Jeol, Tokyo, Japan) prior to any laser irradiation at magnifications of 25X, 60X, 100X and 500X.

### Fixed parameters

As much as is feasible, devices parameters and other aspects that could interfere with or alter the results were controlled, as shown in Table 1.

**Table 1:** Fixed parameters applied for each specific laser wavelength under investigation

Laser Type	Mode	Pulse Duration	Frequency	Air	Water	Distance	Time	Tip Diameter
Er, Cr: YSGG 2780 nm	P	0.06 ms	30 Hz	50%	40%	2 mm	10 s	600 μm
Er: YAG 2940 nm	P	0.3 ms	30 Hz	50%	40%	2 mm	10 s	600 μm
Nd: YAG 1064 nm	P	0.3 ms	30 Hz	-	-	2 mm	10 s	300 μm
Diode 940 nm	P	20 ms	25 Hz	-	-	2 mm	10 s	300 μm
Diode 445 nm	P	495 ms	30 Hz	-	-	2 mm	10 s	320 μm

**Fig. 3:**
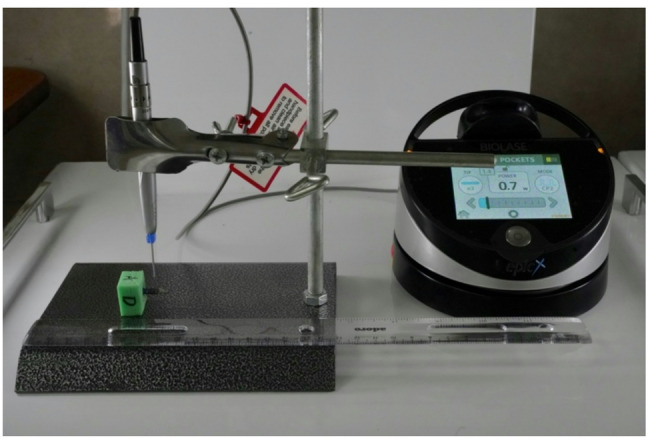
Extension arm and ruler used to fixate the distance between fiber tip and implant surface prior to laser irradiation

1. Pulse mode was used on all laser devices.
2. Frequency was set at 30 Hz for all laser devices with the exception of the Diode (940 nm) laser which was set at 25 Hz as it only allows selection from the device preset parameters.
3. Air and water percentages were fixed at 50% and 40%, respectively, on applicable laser devices with the exception of the Nd: YAG and Diode lasers as they do not include a cooling system in the device.
4. Distance between laser fiber tip and irradiated surface was fixed at 2 mm. This was achieved with the help of an extension arm to which the laser handpiece was attached and measuring of the distance between the fiber tip and implant surface done immediately prior to each laser irradiation (Fig. 3).
5. Exposure time was set at 10 s for each irradiation area.
6. Fiber tip diameter was predetermined with a fiber diameter of 600 microns for Er, Cr: YSGG and Er: YAG lasers, a fiber diameter of 300 microns for Nd: YAG and Diode (940 nm) and a fiber diameter of 320 microns for Diode (445 nm) lasers, as the different laser systems do not accept a common fiber diameter.
7. A four-minute cooldown period was maintained between each laser irradiation to control any latent heat effects that may compile over different irradiations and affect the accuracy of the results.

### Laser irradiation

Irradiation of each area was conducted with the appropriate power setting while maintaining other parameters. Table 2 shows the dosimetry of each power under investigation in combination with different parameters with their corresponding energy density, given that time is fixed at 10 s and distance between fiber tip and implant surface fixed at 2 mm. All irradiations were carried out with the laser tip fixed perpendicular to the implant surface without any vertical or horizontal motion of the implant or the laser handpiece during the irradiation period. All irradiation sessions across the different groups were performed by the same clinician.

**Table 2:** Dosimetry of each power setting under investigation in combination with different parameters

Power (W)	Frequency (Hz)	Pulse Energy (mJ)	Total Energy (J)	Tip Diameter (µm)	Spot Area (cm^2^)	Energy Density (J/cm^2^)
0.75	30	25	7.5	600	0.0028	8.93
1.0	30	33	10	600	0.0028	11.79
1.25	30	42	12.5	600	0.0028	15
1.5	30	50	15	600	0.0028	17.86
0.75	30	25	7.5	300	0.0007	35.71
1.0	30	33	10	300	0.0007	47.14
1.25	30	42	12.5	300	0.0007	60
1.5	30	50	15	300	0.0007	71.43
0.75	25	30	7.5	300	0.0007	42.86
1.0	25	40	10	300	0.0007	57.14
1.25	25	50	12.5	300	0.0007	71.43
1.5	25	60	15	300	0.0007	85.71

### Scanning electron microscope analysis

SEM scans were repeated with the same protocol as the initial scans for assessment and classification, for the new implants to investigate any alterations in the surface topography, and for the failed implants to investigate the efficacy of each wavelength and power setting on removal of the bacterial biofilm. Additionally, Energy Dispersive X-ray Spectroscopy (EDX) surface analysis was conducted at different areas in each laser group, to identify the elements and oxides present in the area under investigation, to confirm the different findings seen on the SEM scans without misinterpretation between surface alterations and possible contaminants present on the implant surface.

### Scoring and classification

A descriptive classification for degree of implant surface alteration was employed to allow comparability and reproducibility of the results in future studies [22].

Alterations of the implant surface were assessed and classified according to the following criteria:

- Class I: No visible signs of surface alteration.
- Class II: Visible alteration or carbonization.
- Class III: Ongoing melting process.
- Class IV: Melting drops can be seen.

Efficacy in removal of biofilm was assessed and classified according to the following criteria:

- No: No visible signs of detachment or removal of biofilm complex.
- Yes: Visible signs of detachment or removal of biofilm complex.

### Statistical analysis

A descriptive analysis was conducted by summarizing the data in terms of frequencies and percentages. Pearson Chi Square test and Fisher’s Exact test were used for comparison between different laser type and laser power intensities. Data were analyzed using IBM SPSS, version 23, Armonk, NY. USA. The significance level was set at *P* <.05.

## Results

### Degree of surface alteration according to laser type

The comparison among different laser groups and across different power intensities revealed significant differences in alterations in surface topography, shown in Table 3.

**Table 3:** Distribution of degree of surface alteration among different laser groups across

Laser Type	Surface Alteration Classification	Power Intensity				*P* value
		0.75 W	1 W	1.25 W	1.5 W	
Er, Cr: YSGG (*n* = 4/intensity)	Class I	4 (100%)	4 (100%)	2 (50%)	2 (50%)	0.238
	Class II	0 (0%)	0 (0%)	2 (50%)	1 (25%)	
	Class III	0 (0%)	0 (0%)	0 (0%)	1 (25%)	
	Class IV	0 (0%)	0 (0%)	0 (0%)	0 (0%)	
Er: YAG (*n* = 4/intensity)	Class I	4 (100%)	3 (75%)	2 (50%)	1 (25%)	0.149
	Class II	0 (0%)	1 (25%)	2 (50%)	3 (75%)	
	Class III	0 (0%)	0 (0%)	0 (0%)	0 (0%)	
	Class IV	0 (0%)	0 (0%)	0 (0%)	0 (0%)	
Nd: YAG (*n* = 4/intensity)	Class I	3 (75%)	2 (50%)	0 (0%)	0 (0%)	0.008*
	Class II	1 (25%)	2 (50%)	4 (100%)	0 (0%)	
	Class III	0 (0%)	0 (0%)	0 (0%)	3 (75%)	
	Class IV	0 (0%)	0 (0%)	0 (0%)	1 (25%)	
Diode 940 (*n* = 4/intensity)	Class I	4 (100%)	3 (75%)	1 (25%)	0 (0%)	0.026*
	Class II	0 (0%)	1 (25%)	3 (75%)	2 (50%)	
	Class III	0 (0%)	0 (0%)	0 (0%)	2 (50%)	
	Class IV	0 (0%)	0 (0%)	0 (0%)	0 (0%)	
Diode 445 (*n* = 4/intensity)	Class I	4 (100%)	2 (50%)	1 (25%)	0 (0%)	0.040*
	Class II	0 (0%)	2 (50%)	2 (50%)	1 (25%)	
	Class III	0 (0%)	0 (0%)	1 (25%)	3 (75%)	
	Class IV	0 (0%)	0 (0%)	0 (0%)	0 (0%)	
*P* value		0.378	0.504	0.477	0.156	

*Statistically significant difference at *P* value < 0.05, *NS* Not significant

### Er, cr: YSGG laser group

**Fig. 4:**
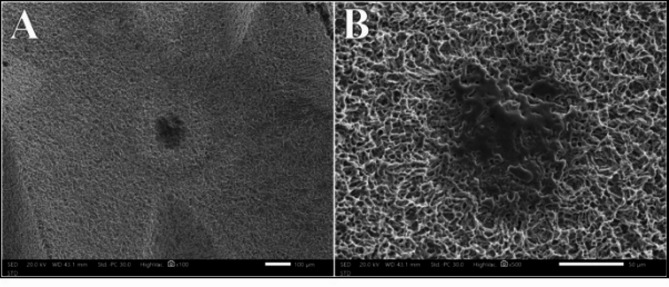
Class III surface alteration after irradiation with Er, Cr: YSGG laser at 1.5 W at magnifications of 100X (A) and 500X (B)

The distribution of surface alteration scores for the Er, Cr: YSGG laser at different powers showed no statistically significant difference (*P* =.238). At 0.75 W and 1 W, 100% of the samples exhibited Class I surface alteration, which decreased to 50% at both 1.25 W and 1.5 W. Class II alterations were observed in 50% of samples at 1.25 W and 25% at 1.5 W. One sample exhibited Class III alteration at 1.5 W power intensity, presenting as a spot of partial melting of the metal on the surface of the implant (Fig. 4).

### Er: YAG laser group

**Fig. 5:**
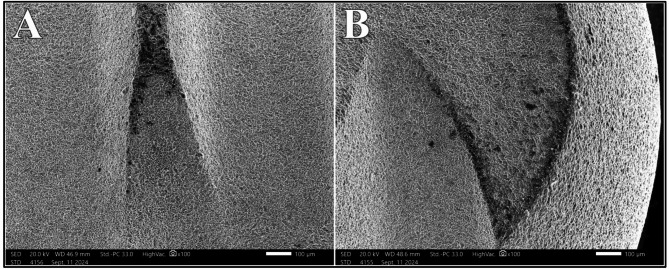
Class II surface alterations after irradiation with Er: YAG laser at 1.25 W (A) and 1.5 W (B) at magnification of 100X

The distribution of surface alteration scores for Er: YAG laser at different power intensities showed no statistically significant difference (*P* =.149). At 0.75 W, 100% of the samples exhibited Class I surface alteration, which decreased to 75% and 50% at 1.0 W and 1.25 W, respectively. At 1.5 W, Class II alterations were observed in 75% of samples; however, no Class III or Class IV alteration were noted across all power intensities under investigation (Fig. 5).

### Nd: YAG laser group

**Fig. 6:**
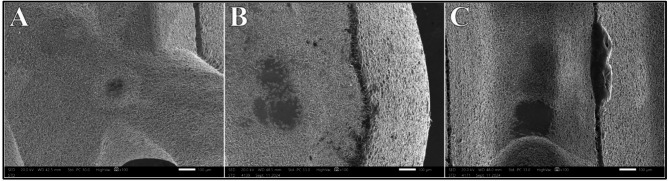
Class III alterations (**A** & **B**) and Class IV alteration (**C**) after irradiation with Nd: YAG laser at 1.5 W at magnification of 100X

Regarding Nd: YAG, distribution of surface alteration scores at different power intensities showed statistically significant difference (*P* =.008). An early evidence of Class II alteration was noted at 0.75 W in 25% of the samples. At 1.0 W, Class II alterations showed a rise to 50%, while at 1.25 W, 100% of the samples displayed Class II alterations. Subsequently using power intensity of 1.5 W caused more severe alterations. Class III alterations comprised 75% of the samples, presenting as areas of partial and complete fusion of the superficial metal layer with loss of microstructure. Class IV alteration constituted the remaining 25% with clear melting drops on the implant surface (Fig. 6).

### Diode 940 nm laser group

**Fig. 7:**
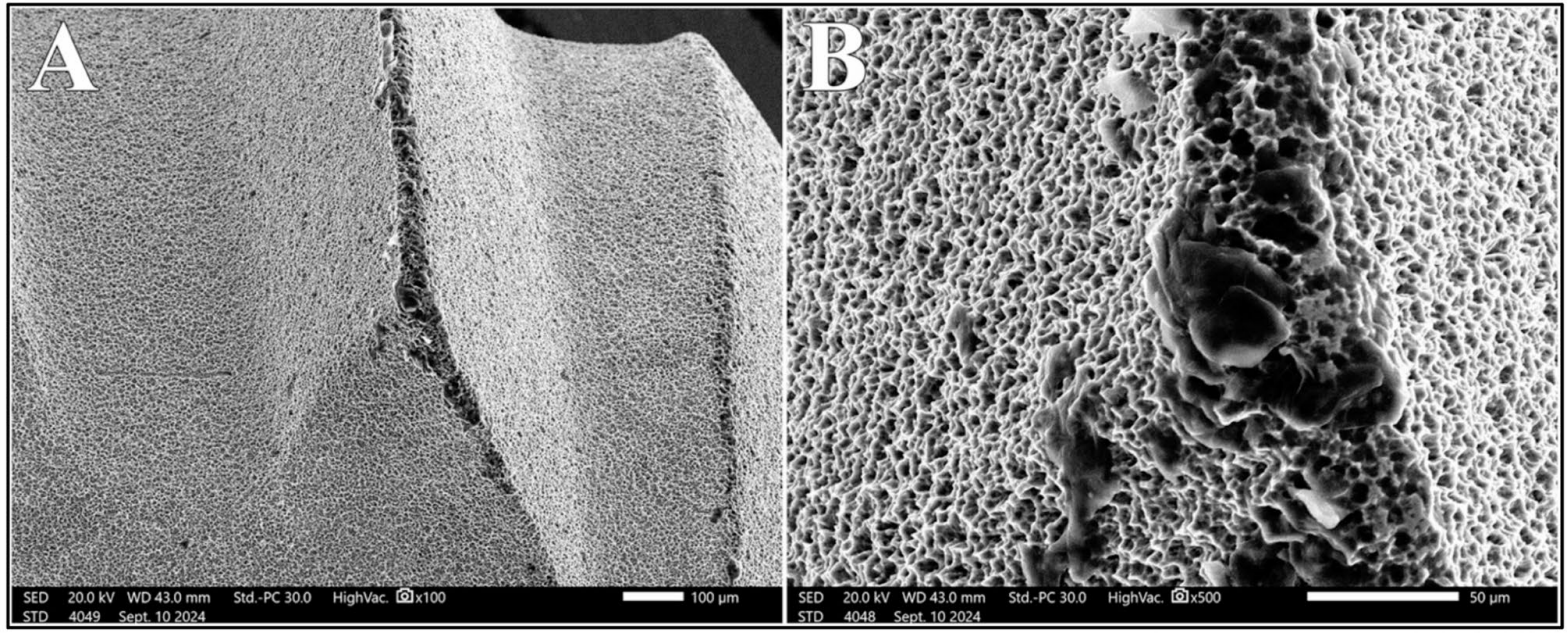
Class III surface alteration after irradiation with Diode 940 nm laser at 1.5 W at magnifications of 100X (**A**) and 500X (**B**)

For the Diode 940 nm, surface alteration scores showed statistically significant difference (*P* =.026). Class I alterations dominated 100% of the samples at 0.75 W. A rise of 25% and 75% of Class II alterations were seen at power intensities of 1.0 W and 1.25 W, respectively. At 1.5 W, Class III alterations involved 50% of the samples; with a corresponding 50% evidence of Class II alterations (Fig. 7).

### Diode 445 nm laser group

**Fig. 8:**
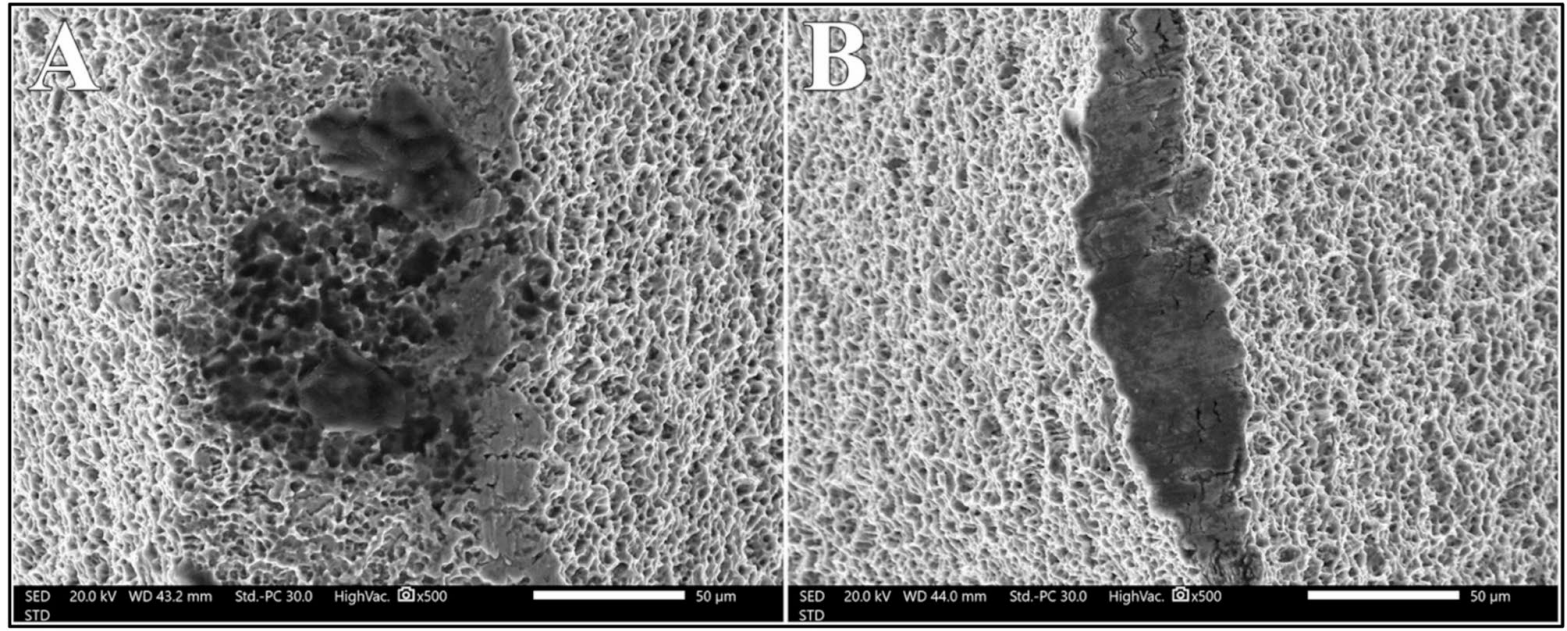
Class III alterations after irradiation with Diode 445 nm laser at 1.25 W (**A**) and 1.5 W (**B**) at magnification of 500X

Comparable to Nd: YAG and Diode 940 nm, the Diode 445 nm showed statistically significant difference in surface alteration scores (*P* =.040). A 100% Class I alteration scoring was noted at 0.75 W; which was reduced to 50% at power intensity of 1.0 W. Upon increasing the power intensity to 1.25 W, a combination of Class I, Class II and early Class III presented on the samples; with Class II being the most frequent at 50%. Additionally, Class III alterations were present on 75% of areas irradiated with 1.5 W (Fig. 8).

### Pairwise comparison between power intensities

Laser groups that have shown statistically significant difference in surface alteration scoring were included in a Pairwise Comparison to evaluate the difference between each specific power setting within the same laser group, shown in Table 4.

The comparison revealed statistically significant difference for all included laser groups at specific power intensities. Nd: YAG and Diode (940 nm and 445 nm) lasers showed statistically significant difference in comparison between using the power intensities of 0.75 W and 1.5 W. In addition, Nd: YAG laser showed statistically significant difference between 1 W and 1.5 W, signifying more power-dependent effects in comparison with the other laser types.

**Table 4:** Pairwise comparison between different power intensities based on laser type

Power	Compared to	*P* value				
		Er, Cr: YSGG	Er: YAG	Nd: YAG	Diode 940	Diode 445
0.75 W	1 W	NS	NS	1.00	1.00	1.00
	1.25 W	NS	NS	0.928	0.514	0.443
	1.5 W	NS	NS	0.009*	0.019*	0.017*
1 W	1.25 W	NS	NS	1.00	1.00	1.00
	1.5 W	NS	NS	0.043*	0.106	0.257
1.25 W	1.5 W	NS	NS	0.492	1.00	1.00

*Statistically significant difference at *P* value < 0.05, *NS* Not significant

### Efficacy of biofilm removal according to laser type

The comparison among different laser groups and across different power intensities revealed significant differences in efficacy of biofilm removal, shown in Table 5.

**Table 5:** Distribution of efficacy in removal of biofilm among the different laser groups across different power intensities

Laser Type	Biofilm Removal Criteria	Power intensity				*P* value
		0.75 W	1 W	1.25 W	1.5 W	
Er, Cr: YSGG (*n* = 4/intensity)	Yes	4 (100%)	4 (100%)	4 (100%)	4 (100%)	NA
	No	0 (0%)	0 (0%)	0 (0%)	0 (0%)	
Er: YAG (*n* = 4/intensity)	Yes	4 (100%)	4 (100%)	4 (100%)	4 (100%)	NA
	No	0 (0%)	0 (0%)	0 (0%)	0 (0%)	
Nd: YAG (*n* = 4/intensity)	Yes	0 (0%)	1 (25%)	4 (100%)	4 (100%)	0.005*
	No	4 (100%)	3 (75%)	0 (0%)	0 (0%)	
Diode 940 (*n* = 4/intensity)	Yes	1 (25%)	1 (25%)	1 (25%)	1 (25%)	1.00
	No	3 (75%)	3 (75%)	3 (75%)	3 (75%)	
Diode 445 (*n* = 4/intensity)	Yes	0 (0%)	1 (25%)	2 (50%)	4 (100%)	0.031*
	No	4 (100%)	3 (75%)	2 (50%)	0 (0%)	
*P* value		0.002*	0.028*	0.031*	0.007*	

*Statistically significant difference at *P* value < 0.05, *NA* Not applicable as data is constant

### Er, cr: YSGG laser group

**Fig. 9:**
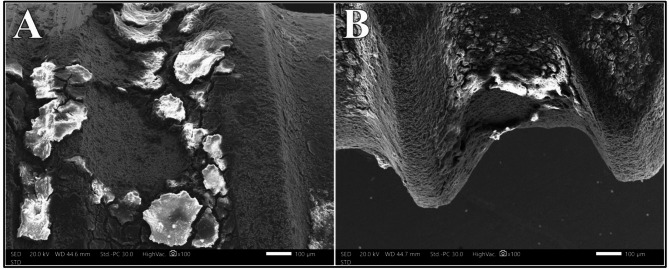
Biofilm complex after irradiation with Er, Cr: YSGG laser at 0.75 W (**A**) and 1.5 W (**B**) at magnification of 100X

The distribution of efficacy in biofilm removal scores for the Er, Cr: YSGG laser at different power intensities showed consistency across all power intensities. Partial or complete disruption of the biofilm complex was achieved on all samples (100%). Additionally, areas demonstrating biofilm ablation became more evident in size and appearance by increasing the power intensity (Fig. 9).

### Er: YAG laser group

**Fig. 10:**
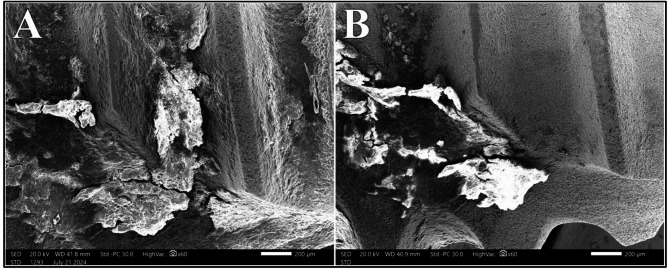
Biofilm complex before laser irradiation (**A**) and after irradiation with Er: YAG laser at 1.5 W (**B**) at magnification of 60X

Similar to the Er, Cr: YSGG group, Er: YAG laser showed consistency in distribution of biofilm removal across all power intensities. In addition to maintaining effectiveness in biofilm removal across all samples (100%), distinct biofilm-free zones were more apparent in areas irradiated with power intensities of 1.0 W or higher (Fig. 10).

### Nd: YAG laser group

**Fig. 11:**
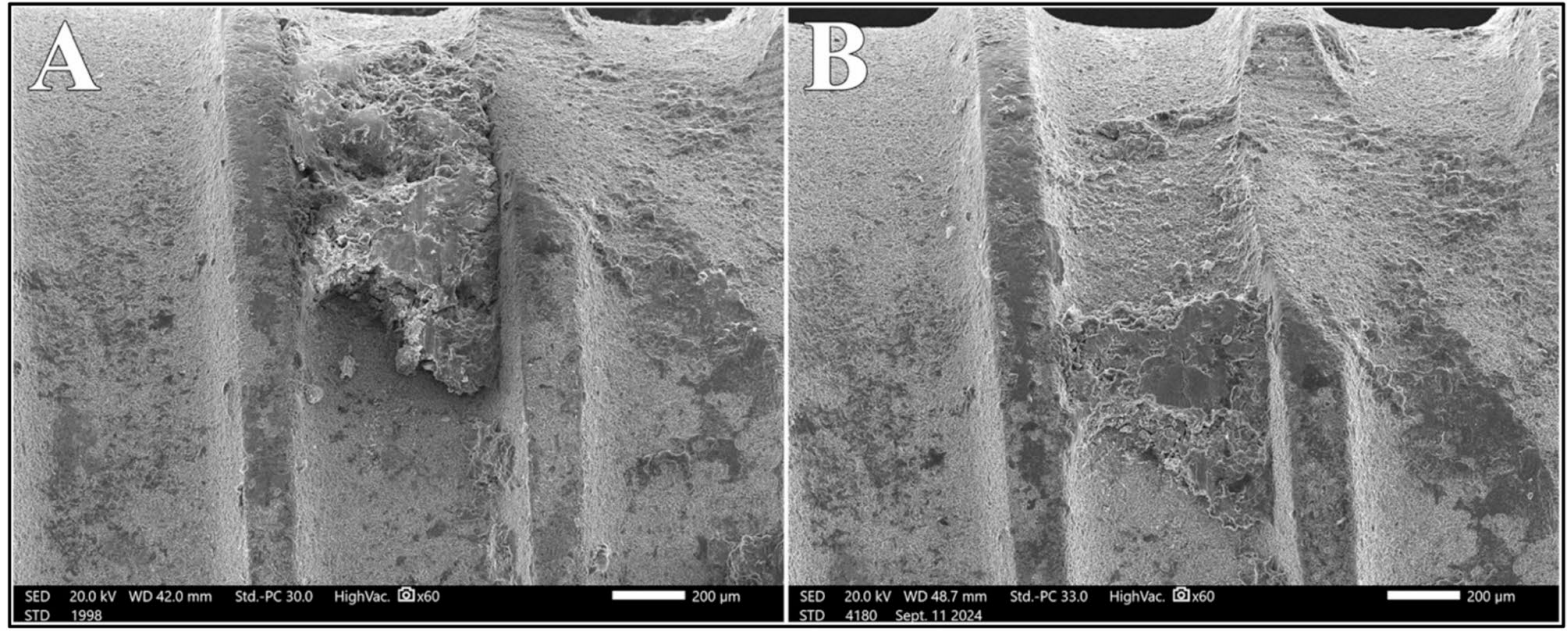
Biofilm complex before laser irradiation (**A**) and after irradiation with Nd: YAG laser at 1.5 W (**B**) at magnification of 60X

The distribution of biofilm removal scores for the Nd: YAG laser at different power intensities showed statistically significant difference (*P* =.005). At 0.75 W, Nd: YAG laser was ineffective in disruption of the biofilm complex (0%). However, increasing the power intensity to 1.25 W and 1.5 W yielded constant positive results in biofilm removal across all samples (100%). Notably, Nd: YAG laser was more efficient in removal of larger complexes in comparison with highly adhering biofilm layers. (Fig. 11).

### Diode 940 nm laser group

**Fig. 12:**
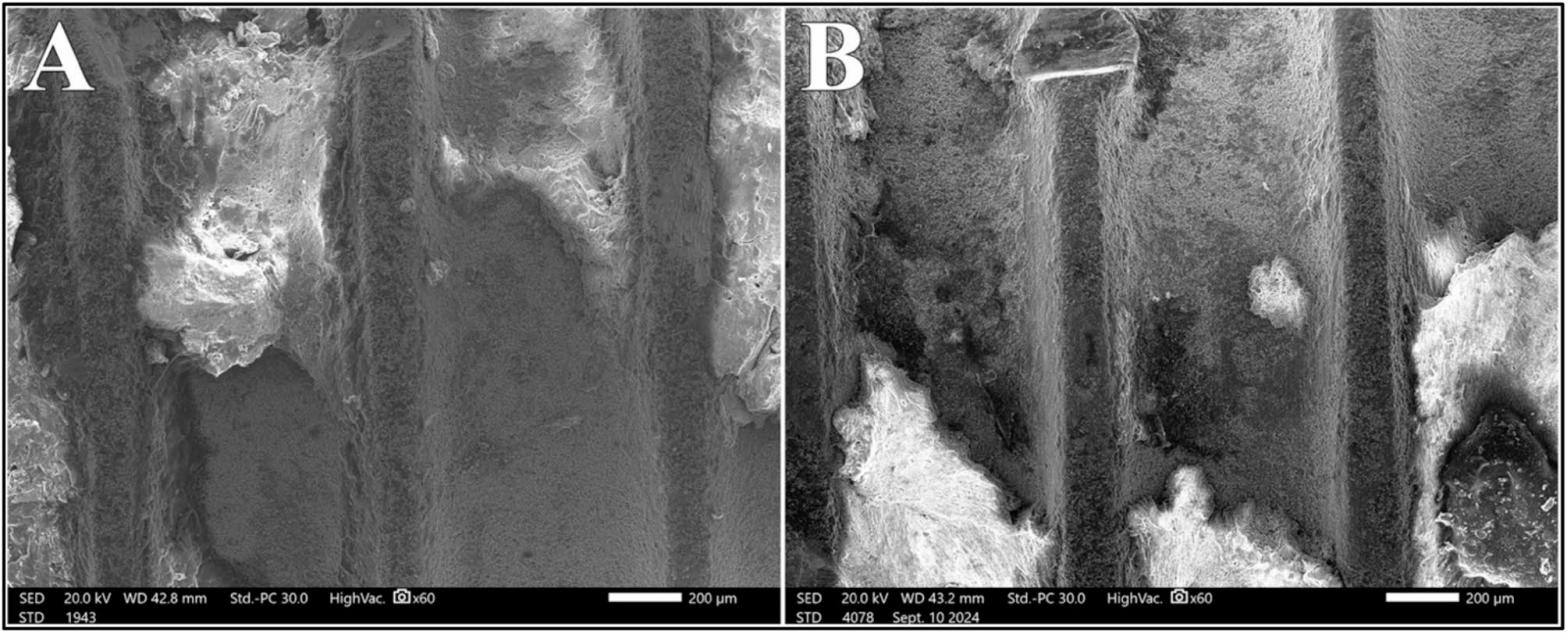
Biofilm complex before laser irradiation (**A**) and after irradiation with Diode 940 nm laser at 1.0 W (**B**) at magnification of 60X

Regarding the Diode 940 nm, no statistically significant difference was found in the distribution of biofilm removal scoring across the different power intensities. (*P* = 1.000). Results remained consistent but with low effectivity, where only 25% biofilm removal was observed across all intensities. Predominantly, Diode 940 nm showed partial dislodgement and reduction in adherence of the biofilm complex from the implant surface rather than complete removal (Fig. 12).

### Diode 445 nm laser group

**Fig. 13:**
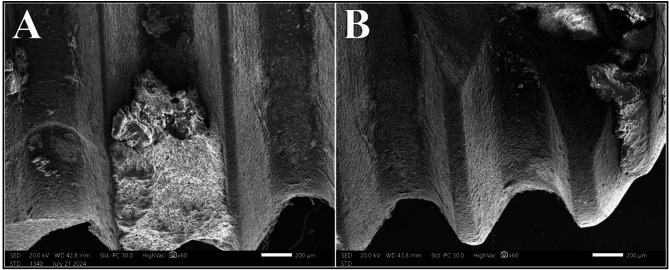
Biofilm complex before laser irradiation (**A**) and after irradiation with Diode 445 nm laser at 1.5 W (**B**) at magnification of 60X

Comparable to the Nd: YAG group, the Diode 445 nm showed statistically significant difference in the distribution of biofilm removal scoring across the different power intensities (*P* =.031). Diode 445 nm was ineffective in removal of the biofilm complex in all samples at 0.75 W (0%). Nonetheless, improvement in efficacy was observed by 25% and 50% at power intensities of 1.0 W and 1.25 W, respectively. Consistent results in removal of the biofilm complex were evident at 1.5 W (100%) (Fig. 13).

### Pairwise comparison between power intensities

Laser groups and power intensities that have shown statistically significant difference in regards to efficacy in biofilm removal were included in a Pairwise Comparison to evaluate the difference between each laser type based on the power intensity used, shown in Table 6, and between each power intensity based on the type of laser used, shown in Table 7.

**Table 6:** Pairwise comparison between each laser type based on the power intensity used

Laser Type	Compared to	*P* value			
		0.75 W	1 W	1.25 W	1.5 W
Er, Cr: YSGG	Er: YAG	NA	NA	NA	NA
	Nd: YAG	0.029*	0.143	NA	NA
	Diode 940	0.143	0.143	0.143	0.143
	Diode 445	0.029*	0.143	0.429	NA
Er: YAG	Nd: YAG	0.029*	0.143	NA	NA
	Diode 940	0.143	0.143	0.143	0.143
	Diode 445	0.029*	0.143	0.429	NA
Nd: YAG	Diode 940	1.00	1.00	0.143	0.143
	Diode 445	NA	1.00	0.429	NA
Diode 940	Diode 445	1.00	1.00	1.00	0.143

*Statistically significant difference at *P* value < 0.05, *NA* Not applicable as data is constant

Both comparisons revealed statistically significant difference for specific laser groups and power intensities. The comparison of Er, Cr: YSGG with Nd: YAG and Diode (445 nm) lasers revealed statistically significant difference at power intensity of 0.75 W (*P* =.029). Er: YAG laser showed similar results in comparison with Nd: YAG and Diode (445 nm) lasers at 0.75 W (*P* =.029). Moreover, Nd: YAG laser showed statistically significant difference in effect between using the power intensity of 0.75 W in comparison to 1.25 W and 1.5 W (*P* =.029). Similarly, Diode (445 nm) laser showed statistically significant difference between using the power intensity 0.75 W in comparison to 1.5 W (*P* =.029).

**Table 7:** Pairwise comparison between each power intensity based on laser type

Power	Compared to	*P* value				
		Er, Cr: YSGG	Er: YAG	Nd: YAG	Diode 940	Diode 445
0.75 W	1 W	NA	NA	1.00	NS	1.00
	1.25 W	NA	NA	0.029*	NS	0.429
	1.5 W	NA	NA	0.029*	NS	0.029*
1 W	1.25 W	NA	NA	0.143	NS	1.00
	1.5 W	NA	NA	0.143	NS	0.143
1.25 W	1.5 W	NA	NA	NA	NS	0.429

*Statistically significant difference at *P *value < 0.05, *NA* Not applicable as data is constant, NS: Not significant

## Discussion

This study aimed to evaluate the effect of using different wavelengths and power settings of dental laser on surface topography of new SLA titanium implants, and to investigate the efficacy of each specific wavelength and power of laser irradiation on removal of the biofilm complex from the surface of failed implants. The null hypothesis was partially rejected as no differences were found regarding efficacy in biofilm removal from the implant surface in the Er, Cr: YSGG, Er: YAG and Diode (940 nm) groups using different power settings; however, significant differences were found regarding surface alteration and efficacy in biofilm removal using different power settings across all other laser groups. Both assessments were conducted by descriptive analysis and comparison of SEM images prior to and following laser irradiations. Results showed that both Er, Cr: YSGG and Er: YAG lasers caused minimal to no alterations in surface topography across the different power intensities. Nd: YAG and Diode lasers showed more evident alterations at high power intensities; with Nd: YAG resulting the most prominent damage to the implant surface. Regarding efficacy of removal of biofilm, Er, Cr: YSGG and Er: YAG lasers consistently exhibited positive results across all different power intensities under investigation. In comparison, Nd: YAG and Diode lasers showed inferior efficacy in biofilm removal at low power intensities with significant power-dependent improvements.

To select appropriate laser types and parameters for the present study, results and considerations from former clinical and experimental studies were reviewed to define suitable settings for decontamination of SLA titanium implants [13, 14, 22–26]. As much as is feasible, devices parameters and other aspects that could interfere with or alter the results were controlled. However, it is important to highlight that using different fiber tip diameters results in different energy densities, where a larger fiber tip diameter results in a wider exposed area to laser irradiation and less thermal effects in comparison with a smaller one. In the present study, fiber tip diameters recommended by previous studies for each specific type of laser were reviewed [14, 22, 24, 26]. Unlike related in vitro studies where titanium disks were used, titanium implants were used in the present study to provide a more accurate demonstration of laser-target interaction with the distinct macro and micro surface topography of implants [20, 23, 24]. However, it is important to note that the specific macro anatomy of each irradiation area can result in varying beam-to-surface angles, which may affect the laser energy transfer to the implant surface.SEM analysis was conducted for interpretation and analysis of the results as it allows repeatability of the methodology and comparability of results with analogous previous and future studies. In the present study, different magnifications (25X, 60X, 100X and 500X) were employed for different purposes; where magnification of 25X was used for all samples for general assessment of the irradiation area, magnifications of 60X and 100X were used to analyze findings in comparison with immediately adjacent zones, and magnification of 500X was used to assess the nature of the findings on a microstructural level. Additionally, EDX analysis was employed at some areas to identify the elements and oxides present to avoid misinterpretation of the different findings; where high carbon peaks indicate surface contamination while oxygen signals could denote that the surface was oxidized as a result of laser irradiation [27].

Different laser types and protocols have proven to be safer and more efficient, in comparison with other types. Including the present study, researches on Erbium lasers indicate consistent superiority in implant decontamination with low findings of negative impacts on the implant surface. Results from an in vitro study evaluating the effect of Er, Cr: YSGG laser on implant surface exhibited minimal reduction in the implant surface roughness without melting when used at power intensities of 1.5 W and 2.5 W in constant motion around the implant [22]. In the present study, scores were comparable with a 25% prevalence of Class III alteration (ongoing melting process) at 1.5 W presented; which could be the result of fixation of the laser tip over the same area during the irradiation process. This lack of movement causes accumulation of thermal energy beyond the metal thermal capacity; which was done in the present study to assess the maximum potential damage that could be induced by laser irradiation [28]. Another study focusing on the surface modification and cell adhesion using Er, Cr: YSGG laser showed minimal to no surface alterations using power intensity of 1.5 W and frequency of 30 Hz with constant horizontal movement [23]. In addition, a significant increase in numbers of attached fibroblasts and osteoblasts was noted following irradiation, demonstrating the diversity of benefits attainable from Er, Cr: YSGG laser. Comparably, a study on laser-assisted regenerative surgical therapy for peri-implantitis using Er: YAG showed improvements in terms of clinical parameters [13]. The use of Er: YAG laser at 50 mJ per pulse with 20 to 25 Hz produced significantly higher PD reduction in comparison with mechanical debridement alone [13, 14]. Clinical signs are indicative of the degree of peri-implantitis; hence, a significant clinical and radiographic improvement is suggestive of the amount of decontamination achieved by laser [29]. On the other hand, an in vitro study on the effect of Er: YAG laser on surface roughness showed that using energy levels exceeding 140 mJ/pulse at 10 Hz, in spite of constant movement of the laser tip during irradiation, could induce melting of SLA-treated implant surfaces [30]. In the present study, lower pulse energy levels, not exceeding 60 mJ, were delivered over a higher frequency of 30 Hz with no movement during irradiation causing minimal to no surface alterations. The comparison between the studies highlights the benefit of energy distribution showing that using lower pulse energies can be sufficient in promoting peri-implant health without altering the implant surface.

Studies evaluating the use of Nd: YAG laser were found to be limited concerning the fact that Nd: YAG laser may provoke damage on the implant surface due to its high penetration potential [20]. Nonetheless, inclusion of Nd: YAG laser in the study seemed necessary to verify the potential positive and negative impacts of its use, in comparison with other types of lasers. One study on surgical treatment of peri-implantitis using a combined Nd: YAG and Er: YAG laser approach produced significant reduction in the BOP sites [25]. However, it has to be underscored that in the study, Nd: YAG laser was employed only for deep tissue decontamination and biomodulation with avoidance of contact with the implant surface, which makes it difficult to identify the exact effect induced by each laser type separately. T. Abduljabbar et al. studied the effect of Nd: YAG laser-assisted non- surgical mechanical debridement on clinical and radiographic peri-implant inflammatory parameters [21]. Results showed that, at 3-month follow-up, scores of PI, BOP and PD were higher among patients in the laser group compared with the control group. At 6-month follow-up, the scores were comparable, with no statistically significant different in peri-implant crestal bone loss in both groups at all time intervals. In the present study, Nd: YAG laser was only able to remove larger biofilm complexes at high power intensities but did not achieve complete cleaning of the area, which could relate to the short-term improvements seen amongst clinical studies, yet more comprehensive studies including microbiological and surface analysis measures is mandatory to confirm these reports. In regards to surface alteration, three titanium disks with different surface treatment were used in a study to determine the effect of Nd: YAG laser on the metallic surface [24]. The SEM examination demonstrated extensive melting in all of the Nd: YAG laser irradiated areas with pulse powers of 40 mJ, 80 mJ and 120 mJ. Comparably, in the present study, using Nd: YAG laser with pulse power of 50 mJ caused partial and complete melting of the metal with visible melting drops. These results validate that Nd: YAG laser presents the risk of inducing significant damage to the implant surface, consequently affecting its osseointegration potential.

Regarding Diode lasers, studies have shown controversy considering both its effect on implant surface topography and efficacy in implant decontamination [18, 26, 31, 32]. In a study on clinical and biochemical outcomes of laser treatment of peri-implantitis, Diode (940 nm) laser was placed parallel to the implant surface and was used in continuous wave mode at 0.8 W with constant apico-coronal and mesial-distal directions for a total of 30 s. Results showed clinical improvement in treatment of peri-implantitis, however, no significant differences in tissue inhibitor metalloproteinase-1 or matrix metalloproteinase-9 levels between the Diode and control groups [32]. In the present study, Diode (940 nm) laser offered only 25% biofilm removal across all power intensities. The comparison of these results implies possible but low clinical effectivity of Diode laser, with higher risk of damage to the implant surface and surrounding tissue accompanying fixation of the laser fiber tip. Another study was conducted on the effect of altering the power of Diode (940 nm) laser on the hydrophilicity, surface topography, and chemical composition of SLA-treated titanium implants. Considerable surface alterations and reduction in hydrophilicity were observed at 3 W followed by the 2 W group, with the least changes observed in association with the 1 W group. Correlated with the present study, minimal alterations were seen up 1.25 W power intensity, with notable melting of the metal surface at 1.5 W, highlighting the different power-sensitive outcomes of using Diode lasers in implant surface modification [27]. As for the Diode (445 nm) laser, a study assessed its thermal effect on five dental implant systems [26]. Results showed temperature increases of more than 10 °C at or above the 0.8 W power level working in CW mode for 5 s and in pulsed mode at 3 W for 20 s with 10% duty cycle. The study has also indicated, through SEM analysis, that no notable surface alterations were noted on the surface of the implants when laser was used in non-contact mode. In comparison with the current study, where 50% DC was employed for all power settings, evident surface alterations were noted at power intensities exceeding 1.0 W. This can be the result of longer pulse durations accompanying higher duty cycles, where rest periods between pulses are considerably shorter. Additionally, in a clinical context, if the critical temperature increase of 10 °C is exceeded, the vitality of surrounding bony and soft tissue structures could be affected [33]. These findings indicate that using lower power intensities in pulse mode results in less potential damage to the implant surface and the surrounding soft and hard tissue. Another SEM evaluation of thermal effects produced by Diode (445 nm) laser on implant surfaces resulted that using power intensities of 2 W and 3 W in continuous mode at 1 mm distance from the implant caused notable damage on the titanium surfaces. However, results suggest that a power intensity of 0.5 W in pulsed emission mode could be used in the treatment of peri-implantitis as no implant surface alterations were detected. These findings correspond with results from the present study, in terms of surface topography alterations, where notable surface alterations were only observed in power intensities of 1.25 W and 1.5 W. On the other hand, in the present study, using power intensities of 0.75 W and 1.0 W showed clear but low efficacy in removal of the biofilm complex [34]. In another study evaluating alterations of SLA titanium implants following laser irradiation, Diode (940 nm) laser was fixed at 2 mm distance from the implant surface and irradiated for a period of 15 s. Results showed mean temperature increases of 55.2 ^◦^C, 61.8 ^◦^C and 78.4 ^◦^C when laser was used at 1 W, 2 W and 3 W, respectively [35]. These results emphasize that fixed exposure of irradiation may increase implant temperature which will adversely affect the supporting structures. Therefore, non-fixed irradiation (apico-coronal or mesial-distal motion) at lower power intensities is recommended to decrease thermal effects.

Limitations of this study included the small sample size and the lack of true clinical environmental factors. Additionally, the present study employed a descriptive analysis of the SEM images. Further multi-center, large-scale, randomized-controlled clinical studies are recommended using other methods such as microbiological or surface analysis measurements.

## Conclusions

Within the limitations of this study, it was concluded that: (1) Er, Cr: YSGG and Er: YAG lasers are safe to use in decontamination of dental implants without causing notable surface alterations. (2) Diode (940 nm) laser can be used at low power intensities without causing detrimental surface alterations. (3) Nd: YAG and Diode (445 nm) lasers can induce more evident surface damage compromising the osseointegration potential of the implant.

Regarding efficacy of removal of biofilm, it was concluded that: (4) Er, Cr: YSGG and Er: YAG lasers present superior implant decontamination potential. (5) Diode (940 nm) laser presents potential in biofilm removal at safe levels of power intensities. (6) Nd: YAG and Diode (445 nm) lasers show high risks to the implant surface with low efficacy in disruption of the biofilm complex.

## Data Availability

The datasets used and analysed during the current study are available from Paul Ashraf Sedrak (corresponding author) on request.

## Abbreviations

Er: YAG Erbiumdoped Yttrium Aluminum Garnet
Nd: YAG Neodymiumdoped Yttrium Aluminum Garnet
SEM: Scanning Electron Microscope
GI: Gingival Index
PI: Plaque Index
PD: Pocket Depth
BOP: Bleeding on Probing
CAL: Clinical Attachment Level
SLA: Sandblasted Large Grit AcidEtched
EDX: Energy Dispersive Xray Spectroscopy
